# Waste-to-Treasure: Nanozyme-Integrated Photocatalytic Microneedles Reconfigure Diabetic Burn Wounds via Endogenous Glucose-to-H_2_ Conversion and Adaptive Hydrogel Scaffolding

**DOI:** 10.34133/research.1403

**Published:** 2026-09-23

**Authors:** Pei Wang, Xiaofeng Wang, Yating Yang, Zhen Gao, Xiaoxi Lin, Xiansong Wang

**Affiliations:** ^1^ Department of Plastic and Reconstructive Surgery, Shanghai Key Laboratory of Tissue Engineering, Shanghai Ninth People’s Hospital, Shanghai Jiao Tong University School of Medicine, Shanghai 200011, China.; ^2^ Excellent Science and Technology Innovation Group of Jiangsu Province, College of Environmental Science, Nanjing Xiaozhuang University, Nanjing 211171, China.

## Abstract

The recalcitrance of diabetic burn wounds is rooted in a hostile microenvironment characterized by a pathological surplus of glucose, advanced glycation end products (AGEs), and reactive oxygen species. Standard enzymatic and photocatalytic interventions are often limited by instability or the toxic accumulation of hydrogen peroxide (H_2_O_2_), leading to secondary oxidative injury. In this study, we developed a metabolism-driven microneedle platform (MN-PCN) by integrating photocatalytic Pt@Pd-PCN222-NH_2_ (PCN) into a self-adjusting, antimicrobial, and antioxidant hydrogel matrix featuring highly moisture-resilient adhesion. Adopting a waste-to-treasure logic, the system exploits endogenous glucose as a primary reductant to facilitate continuous hydrogen (H_2_) evolution, effectively suppressing the AGEs/receptor for AGEs signaling axis. Critically, the MN-PCN system actively scavenges catalytic H_2_O_2_ byproducts and environmental reactive oxygen species through the synergistic catalase-mimetic activity of the PCN sites and the antioxidant hydrogel matrix, thereby circumventing oxidative damage. Simultaneously, the microneedle architecture undergoes an in situ transition into a protective hydrogel upon contact with wound exudate, establishing a physical barrier that minimizes infection and fosters cellular infiltration. By neutralizing inflammatory circuit and accelerating vascularization, MN-PCN restores both physiological and structural equilibrium. This integrated strategy provides a sophisticated framework for complex wound management through systemic, metabolism-driven microenvironmental reconfiguration.

## Introduction

Diabetic burn wounds represent a formidable clinical challenge, characterized by a self-reinforcing pathological microenvironment that severely hinders the natural regenerative process [1–4]. Under the systemic background of chronic hyperglycemia, these wounds are inundated with an abundance of metabolic waste, specifically advanced glycation end products (AGEs), excessive reactive oxygen species (ROS), and high levels of peroxides [5–7]. These pathological byproducts are not merely passive markers of disease but are synergistically coupled to form a vicious cycle. This feedback loop drives the persistent accumulation of AGEs and the activation of their receptors (receptors for AGEs [RAGEs]), triggering sustained pro-inflammatory cascades and paralyzing the angiogenic potential of vascular endothelial cells [8,9]. Furthermore, compromised barrier function and impaired neural perception in diabetic patients substantially increase the risk of secondary infections, particularly those involving *Staphylococcus aureus* [10–13]. Consequently, the wound is locked in a state of chronic stagnation.

Traditional therapeutic modalities, including surgical debridement and conventional wound dressings, frequently fail to address the underlying biochemical complexity of diabetic burn wounds [14–16]. Although biological enzymes such as glucose oxidase and catalase (CAT) have been investigated to restore metabolic homeostasis [17,18], their clinical utility is restricted by inherent enzymatic instability, rapid denaturation in the hostile wound bed, and the difficulty of precise dosage control [19–24]. Recently, photocatalytic strategies have emerged as a promising alternative for metabolic intervention. For instance, hydrogen-incorporated titanium oxide nanorods have been developed to achieve simultaneous glucose depletion and hydrogen generation [25]. However, this approach faces a critical selectivity bottleneck, since the photogenerated holes responsible for oxidizing glucose often lead to the unintended accumulation of hydrogen peroxide (H_2_O_2_) [26,27]. This secondary oxidative stress, if not promptly neutralized, can inflict further damage on fragile nascent tissues, thereby undermining the therapeutic benefits of hydrogen therapy.

To overcome these limitations, our previous research identified various polyphenols and nanoparticles with H_2_O_2_-scavenging capabilities [28–32], yet the development of an active catalytic system for synchronized metabolic reconfiguration remains a priority. Palladium (Pd) has demonstrated exceptional CAT-mimetic activity, enabling the efficient decomposition of H_2_O_2_ while simultaneously facilitating local AGE degradation and glucose depletion [33,34]. Similarly, platinum (Pt) is also noted for its superoxide dismutase (SOD)-, CAT-, and peroxidase-like properties [35]. Building upon recent advancements in single-atom site engineering [36], the integration of Pt and Pd within a porphyrinic metal–organic framework (PCN222) provides a robust platform for visible-light-driven hydrogen evolution [37,38]. The rational design of Pt/Pd dual sites within PCN222 constitutes a nanozyme engineering strategy that confers cascade SOD-, CAT-, and peroxidase-like activities, enabling sequential ROS neutralization and glucose oxidation. This multi-site nanozyme framework thus drives sustained hydrogen production from pathological glucose while avoiding peroxide accumulation. However, the integration of such catalytic systems into adaptive platforms for diabetic wound reconfiguration remains insufficiently explored [39,40].

Beyond biochemical modulation, the repair of extensive tissue defects necessitates a supportive physical scaffold. Conventional clinical materials, such as acellular dermal matrices, lack the capacity for active microenvironmental regulation. Although advanced hydrogel systems have recently progressed chronic wound management through innovative modalities [41–44], comprehensively reforming the hostile diabetic microenvironment remains a formidable and unfulfilled challenge. Similarly, our previously developed poly(γ-glutamic acid) (γ-PGA)/poly-L-lysine (ε-PLL) (PP) hydrogels [45], despite exhibiting notable antibacterial and antioxidant properties [30,46], still lack the dynamic self-adaptability required for irregular diabetic burn surfaces. This structural mismatch often results in interfacial gaps between the dressing and the wound bed, creating mechanical stress that restricts fibroblast (FB) migration and cellular ingrowth. Consequently, a truly effective therapeutic system must integrate physical–biochemical signals within a platform capable of deep-tissue penetration and seamless biointerfacial adaptation [47–49].

**Fig. 1.**
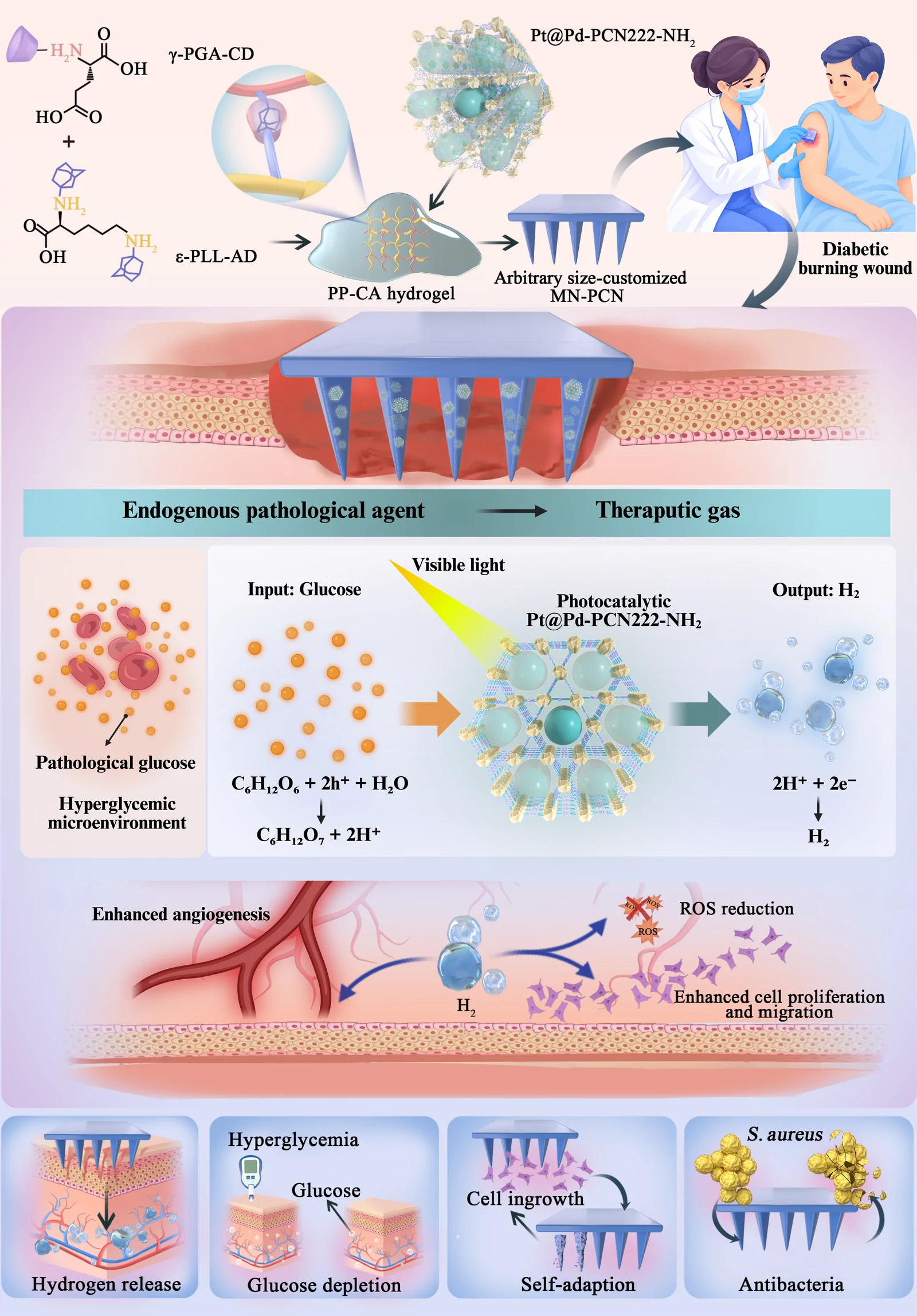
Fabrication and therapeutic mechanism of MN-PCN microneedles. Schematic illustrating the synthesis of MN-PCN microneedles by integrating photocatalytic Pt@Pd-PCN222-NH_2_ (PCN) within a poly(γ-glutamic acid)-cyclodextrin (γ-PGA-CD) and poly-L-lysine-adamantane (ε-PLL-AD) cross-linked (PP-CA) hydrogel. The diagram highlights the therapeutic functions of MN-PCN, including hydrogen generation, glucose depletion, advanced glycation end product (AGE) reduction, and antimicrobial activity for improved diabetic burn wound healing.

In this study, we report a bio-integrated photocatalytic microneedle system (MN-PCN) designed for the systemic reconstruction of the diabetic burn wound microenvironment, based on a hydrogel matrix (PP-CA) formed by γ-PGA-cyclodextrin (γ-PGA-CD) and ε-PLL-adamantane (ε-PLL-AD) (Fig. 1). This platform utilizes 500-μm microneedles to deliver Pt@Pd-PCN222-NH_2_ (PCN) catalytic centers directly into deep tissue layers. Under visible light irradiation, the system orchestrates a sophisticated metabolic transformation: the photogenerated holes oxidize pathological glucose into gluconic acid, a process that effectively suppresses charge recombination and ensures sustained hydrogen evolution. Through this energy-harvesting mechanism, MN-PCN disrupts the AGEs/RAGE-mediated inflammatory cascade at the molecular level and neutralizes oxidative stress via in situ generated hydrogen [50,51]. When combined with PP-CA hydrogel characterized by superior wet-tissue adhesion and self-healing properties, the system simultaneously provides antimicrobial protection and facilitates rapid cellular ingrowth [52]. This integrated strategy of converting pathological waste into regenerative power establishes a new paradigm for the clinical management of complex diabetic burn wounds.

## Results and Discussion

### Fabrication and characterization of PCN and MN-PCN

**Fig. 2.**
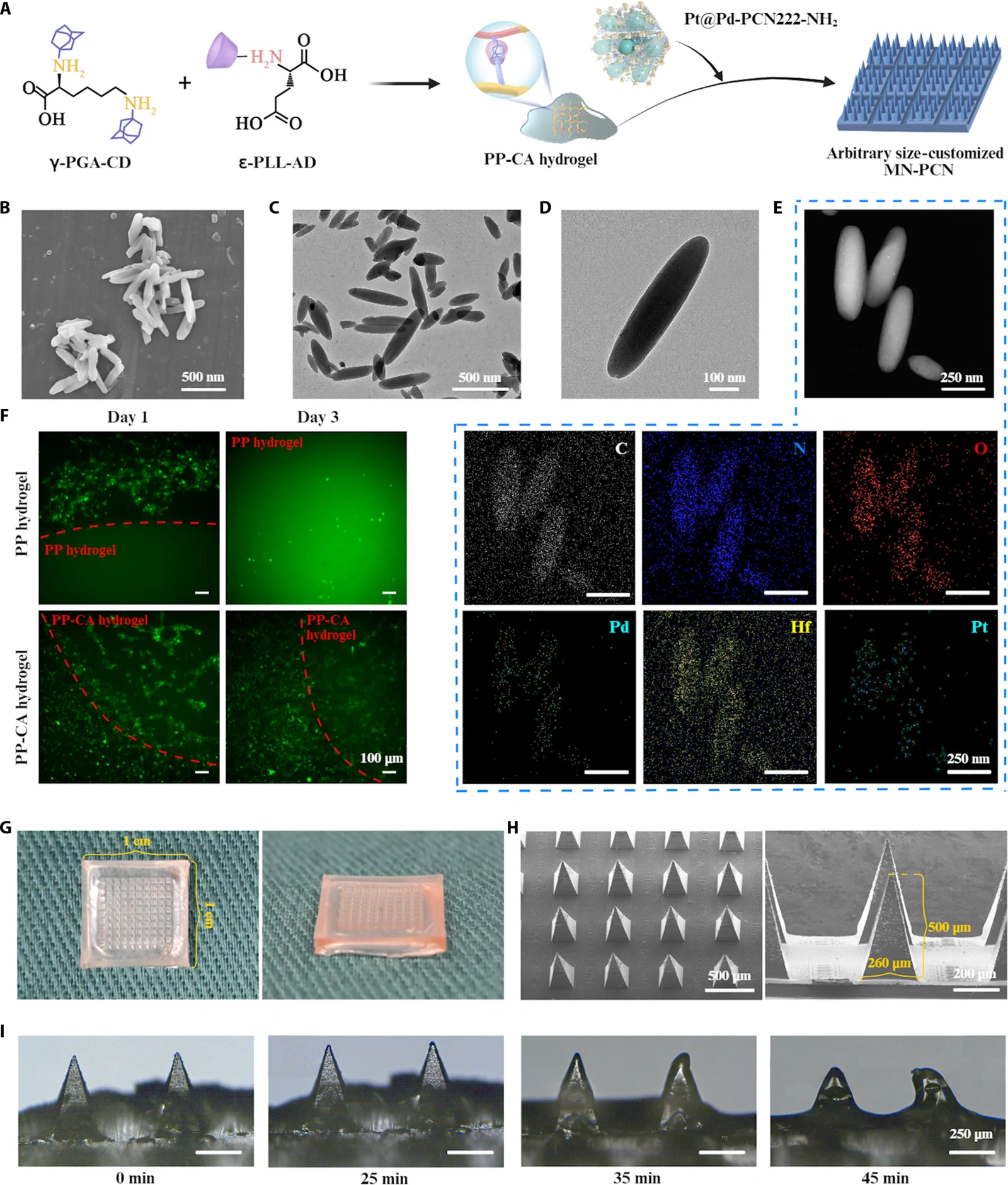
Characterization of Pt@Pd-PCN222-NH_2_ (PCN) nanoparticles and MN-PCN microneedles. (A) Schematic of MN-PCN fabrication. (B to D) Scanning electron microscopy (SEM) and transmission electron microscopy (TEM) images of PCN nanoparticles, confirming their rodlike structure and morphology. (E) Elemental mapping showing the uniform distribution of Pt and Pd in PCN. (F) Enhanced self-adaptability of the PP-CA hydrogel. (G) Photographs of MN-PCN microneedle patches. (H) SEM images of MN-PCN microneedles. (I) In vitro biodegradability of MN-PCN at 25 °C and 75% humidity.

To establish a platform for microenvironment reconfiguration, a bio-integrated photocatalytic microneedle system (MN-PCN) was developed by incorporating PCN nanoparticles into a hydrogel matrix (PP-CA) (Fig. 2A). Unlike simple polymer blending, the PP-CA matrix was fabricated via host–guest recognition between γ-PGA-CD and ε-PLL-AD, progressing from precursor solutions to a cohesive supramolecular network. Macroscopic fabrication assays confirmed that this strategy allows for a highly controllable transition from liquid precursors to a solid-state hydrogel (Fig. S1A). Scanning electron microscopy and transmission electron microscopy confirmed the rodlike morphology of the PCN framework and the uniform distribution of Pt and Pd elements (Fig. 2B to D). Elemental mapping validated the uniform dispersion of these catalytic sites, which is essential for ensuring consistent metabolism-driven conversion of glucose to H_2_ across the microneedle interface (Fig. 2E).

The physical performance of the PP-CA matrix was evaluated to confirm its role in reestablishing biomechanical homeostasis. The hydrogel exhibited autonomous and rapid self-healing capabilities, effectively restoring its structural integrity after mechanical compromise as demonstrated by macroscopic observation and indocyanine green staining (Fig. S1B and C). Beyond simple structural recovery, the self-adaptive nature of the supramolecular cross-linking was found to actively modulate the biomechanical interface. Co-culture assays with green fluorescent protein (GFP)-labeled FBs revealed robust and deep cellular ingrowth into the PP-CA matrix over 3 d, significantly surpassing the limited infiltration capacity of nonadaptive PP hydrogels (Fig. 2F and Fig. S1D). This result underscores the capacity of MN-PCN to serve as a dynamic physical–biological integrated platform that facilitates cellular migration and tissue integration.

The MN-PCN system was engineered as a 10 × 10 array, with individual microneedles measuring 260 μm × 260 μm × 500 μm (*W* × *L* × *H*), maintaining precise geometry as confirmed by scanning electron microscopy (Fig. 2G and H). The 500-μm height is specifically designed to bypass the epidermal barrier and establish deep biochemical transmission channels into the compromised diabetic tissue layers. In vitro and in vivo degradation tests confirmed that the microneedle tips dissolve within 45 min, facilitating the sustained release of PCN into the pathological microenvironment for localized metabolic remodeling (Fig. 2I and Fig. S2).

The functional foundation of metabolic remodeling was validated through the simultaneous capture of pathological glucose and generation of therapeutic gas. The MN-PCN system achieved a significant glucose reduction of 2.35 g l^−1^ within 60 min while initiating rapid H_2_ evolution within 3 min (Fig. S1E and F). This process effectively transforms the high-glucose pathological burden of diabetic wounds into a fuel source for hydrogen therapy. Additionally, the integrated ε-PLL component maintained its potent antimicrobial activity, as MN-PCN enlarged the *S. aureus* inhibition zone by 3.58-fold compared to controls (Fig. S1G and H, Supplementary Materials).

Collectively, these results demonstrate the successful development of a system that actively reconfigures the hostile wound environment into a reparative one through the synergy of adaptive physical scaffolding and metabolic energy capture.

### In vitro therapeutic efficacy of MN-PCN

**Fig. 3.**
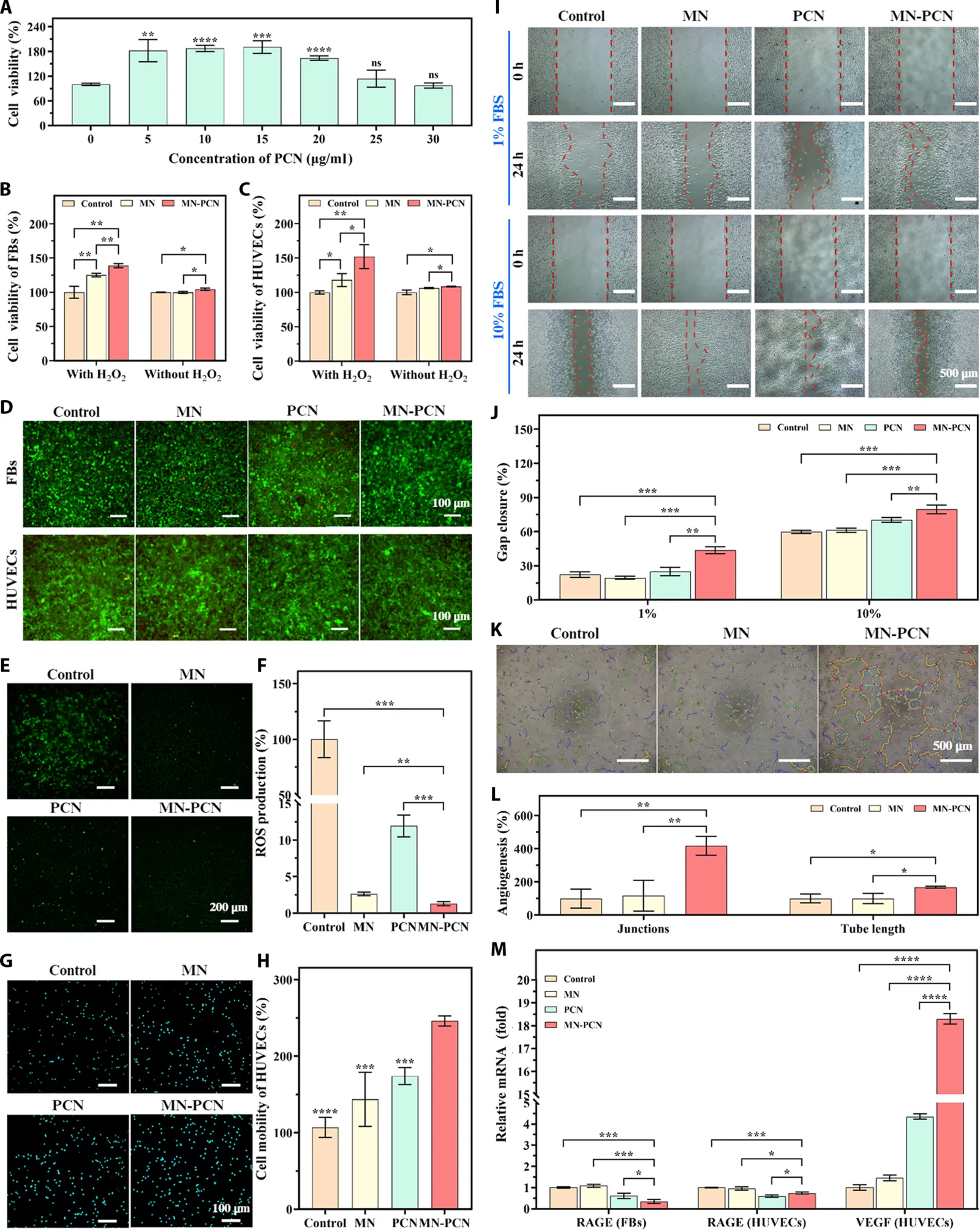
In vitro therapeutic effects of MN-PCN on cell proliferation and migration. (A) Human umbilical vein endothelial cells (HUVEC) viability with varying Pt@Pd-PCN222-NH_2_ (PCN) concentrations for 3 d. (B and C) Viability of HUVECs and fibroblasts (FBs) treated with MN extract, MN-PCN extract, or control, with/without 3 mM H_2_O_2_ exposure. (D) Live/dead staining of HUVECs and FBs after 3-d treatment. (E and F) Reactive oxygen species (ROS) levels in Raw264.7 cells treated with PCN, MN extract, MN-PCN extract, or control, assessed by ROS assay and flow cytometry. (G and H) Transwell assay images and migration quantification of HUVECs. (I and J) Scratch assay images and gap closure quantification of FBs with different treatments. (K and L) Tube formation assay images and quantification of junctions and tube lengths in HUVECs. (M) Messenger RNA (mRNA) levels of receptor for advanced glycation end products (RAGE) and vascular endothelial growth factor (VEGF) in treated FBs and HUVECs. Data represent mean ± SD (*n* = 3); significance: *P < 0.05; **P < 0.01; ***P < 0.001; ****P < 0.0001.

To determine the optimal concentration for systemic reconfiguration, the cytotoxicity of PCN was evaluated on human umbilical vein endothelial cells (HUVECs) using the Cell Counting Kit-8 (CCK-8) assay. Results indicated no significant decrease in cell viability at concentrations below 30 μg ml^−1^, and 15 μg ml^−1^ was identified as the optimal concentration for subsequent experiments (Fig. 3A). Consequently, 15 μg ml^−1^ PCN was used in all subsequent cell experiments. Building on previous studies that demonstrated γ-PGA hydrogel’s antioxidant properties, we hypothesized that the MN-PCN system would exhibit enhanced antioxidant efficacy through the combined effects of H_2_ and γ-PGA.

To assess the cytoprotective effects of this integrated platform, FBs and HUVECs were exposed to 3 mM H_2_O_2_ for 30 min to simulate oxidative damage. Treatment with MN-PCN extract resulted in a 1.39-fold and a 1.52-fold increase in viability for FBs and HUVECs, respectively, compared to untreated controls (Fig. 3B and C). Live/dead cell staining further corroborated these findings, showing enhanced survival in MN-PCN-treated cells (Fig. 3D). The capacity for ROS reduction was quantified using Raw264.7 cells. Flow cytometry revealed that MN-PCN treatment reduced ROS production by 76.77-fold, a significant improvement over the 37.34-fold reduction observed in the MN group (Fig. 3E and F). This enhanced efficacy is attributed to the photocatalytic H_2_ evolution, which neutralizes intracellular ROS and interrupts the oxidative stress cycle.

The regenerative potential driven by biochemical signaling was evaluated through migration and angiogenesis assays. The MN-PCN group demonstrated a 2.30-fold increase in HUVEC mobility over controls and a 1.41-fold increase compared to the PCN group in the Transwell assay (Fig. 3G and H). Similarly, FBs treated with MN-PCN showed a 1.97-fold higher gap closure ratio in complete medium and a 1.33-fold increase in 1% fetal bovine serum (FBS) compared to the control in the scratch assay (Fig. 3I and J). The angiogenic capacity was further quantified using the Matrigel tube formation assay. The MN-PCN extract enhanced junction formation and total tube length by 4.19-fold and 1.68-fold, respectively, compared to controls (Fig. 3K and L). These cellular responses indicate that the platform provides necessary signals for tissue reconstruction.

To elucidate the underlying molecular mechanism of microenvironment remodeling, reverse transcription quantitative polymerase chain reaction (RT-qPCR) was performed. MN-PCN treatment led to a significant down-regulation of RAGE messenger RNA (mRNA) expression while simultaneously up-regulating vascular endothelial growth factor (VEGF) expression in both FBs and HUVECs (Fig. 3M). Primer sequences are listed in Table S1. These findings demonstrate that the system disrupts the metabolic basis of chronic inflammation by suppressing the AGEs/RAGE axis and establishes a proregenerative environment through the up-regulation of angiogenic factors.

In summary, MN-PCN demonstrates marked in vitro therapeutic efficacy by promoting cell proliferation, reducing ROS production, enhancing cell migration, and stimulating angiogenesis. These multifaceted effects position MN-PCN as a promising candidate for the treatment of diabetic burn wounds in vivo.

### In vivo wound healing efficacy of MN-PCN on diabetic burn wounds with or without infection

**Fig. 4.**
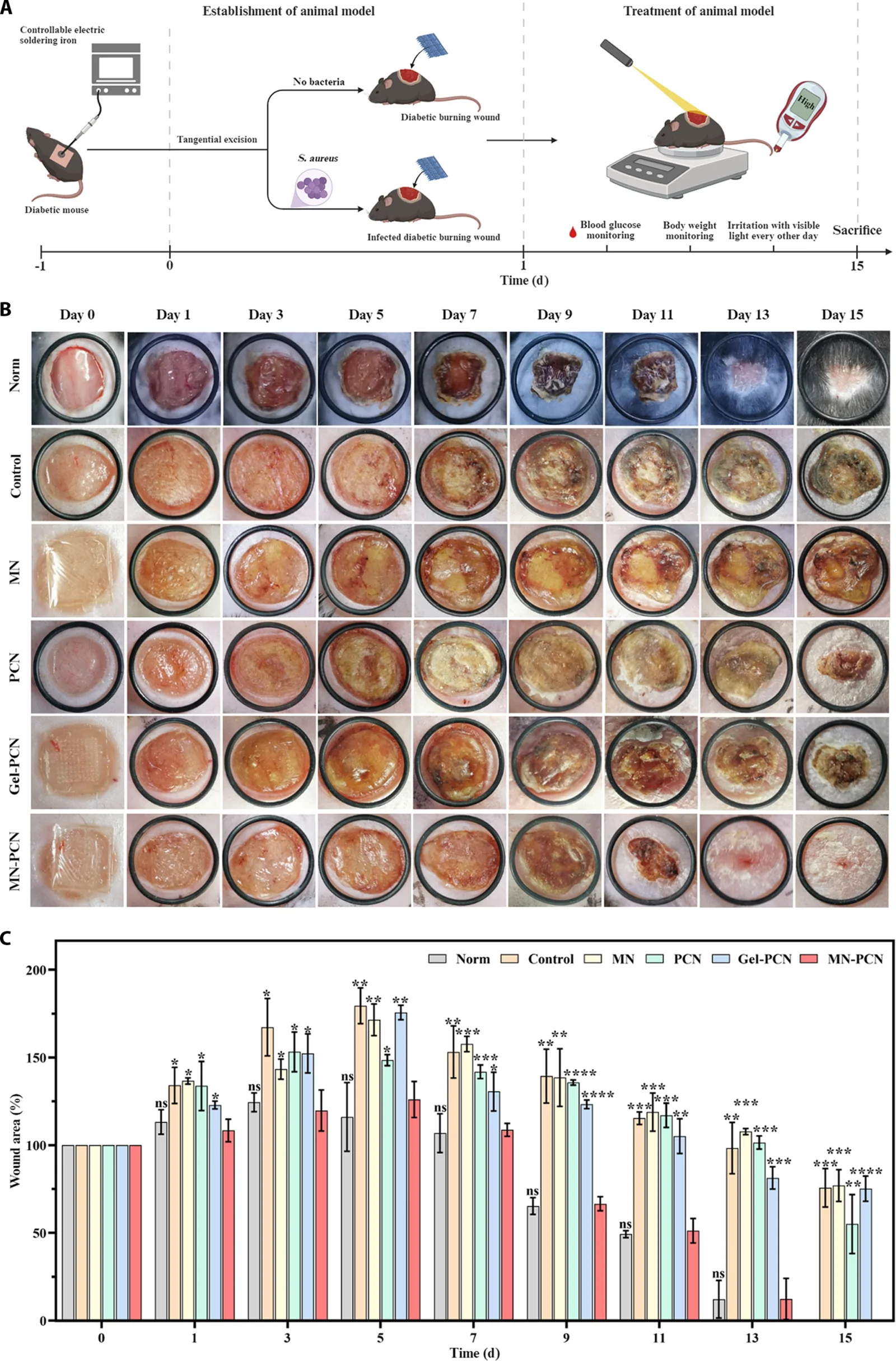
In vivo therapeutic efficacy of MN-PCN on diabetic burn wound healing. (A) Schematic of creating and treating diabetic burn wounds with/without *Staphylococcus aureus* infection. (B) Representative images of BKS (normoglycemic) and diabetic mice with wounds treated with phosphate-buffered saline (PBS), MN, Pt@Pd-PCN222-NH_2_ (PCN) powder, gel-PCN, or MN-PCN over 0, 1, 3, 5, 7, 11, and 15 d. Microneedle patches were left intact until natural detachment. (C) Quantitative analysis of wound area in each treatment group over time. Data represent mean ± SD (*n* = 3); significance: *P < 0.05; **P < 0.01; ***P < 0.001; ****P < 0.0001.

To validate the capacity of MN-PCN for microenvironment reconfiguration, a diabetic mouse model with full-thickness burn wounds was established. Deep burns were created using an electric soldering iron, followed by eschar removal to simulate the clinical pathological state (Fig. 4A). The subjects were randomly assigned to 6 groups: phosphate-buffered saline (PBS; control), MN, PCN powder, gel-PCN, and MN-PCN, with nondiabetic BKS mice (Norm) serving as a physiological baseline. The integration of the system within the wound was initiated by the rapid dissolution of the 500-μm microneedle tips, which established biochemical channels for the delivery of catalytic PCN into deep tissue layers (Fig. S2). Systemic safety was confirmed by histological analysis of major organs and consistent weight gain across groups, demonstrating the biocompatibility of the platform (Fig. S3A and B).

The therapeutic impact of metabolic and physical synergy was evidenced by the wound closure rates (Fig. 4B). Wounds treated with MN-PCN achieved complete closure by day 13, matching the healing rate of the nondiabetic Norm group and significantly outperforming the gel-PCN group (Fig. 4C). This discrepancy between MN-PCN and gel-PCN underscored the necessity of the microneedle physical structure. Without the 500-μm penetration provided by the MN array, the catalytic reconfiguration of the deep-seated pathological microenvironment was severely limited. Histological evaluation via hematoxylin and eosin (H&E) and Masson’s trichrome staining further corroborated this, showing superior epithelial regeneration and highly organized collagen deposition in the MN-PCN group, indicative of a transition from a chronic inflammatory state to a reparative one (Fig. S4A and B).

To address the additional pathological burden of infection, the efficacy of the system was evaluated in *S. aureus*-infected diabetic burn wounds (Fig. 4A). Successful infection was confirmed through bacterial cultures of skin tissues after 48 h (Fig. S5A). By day 7, the MN-PCN group demonstrated potent antimicrobial activity, suppressing bacterial proliferation by 3.58-fold relative to controls (Fig. S5B and C). Despite the exacerbated inflammatory milieu, the MN-PCN system facilitated complete wound closure by day 15 (Fig. S6A). Quantitative analysis revealed that the wound area in the MN-PCN group decreased significantly at each time point relative to those of other groups (Fig. S6B). Histological analysis also revealed robust granulation tissue formation and mature collagen fiber repair (Fig. S7). Throughout the treatment period, MN-PCN-treated mice exhibited stabilized blood glucose levels, suggesting that local glucose depletion at the wound site would not endanger systemic metabolic stabilization (Fig. S5D and E).

The superiority of the MN-PCN platform is rooted in its ability to actively reconfigure the hostile microenvironment. By coupling the physical bypassing of the epidermal barrier with the metabolic conversion of glucose into therapeutic H_2_, the system interrupts the downstream pathological signaling of AGEs/RAGE and restores tissue homeostasis. Collectively, these results confirm that the integrated physical–chemical feedback provided by MN-PCN is essential for accelerating the healing of complex, infected diabetic burn wounds.

### In vivo spatiotemporal catalytic dynamics characterization of MN-PCN

**Fig. 5.**
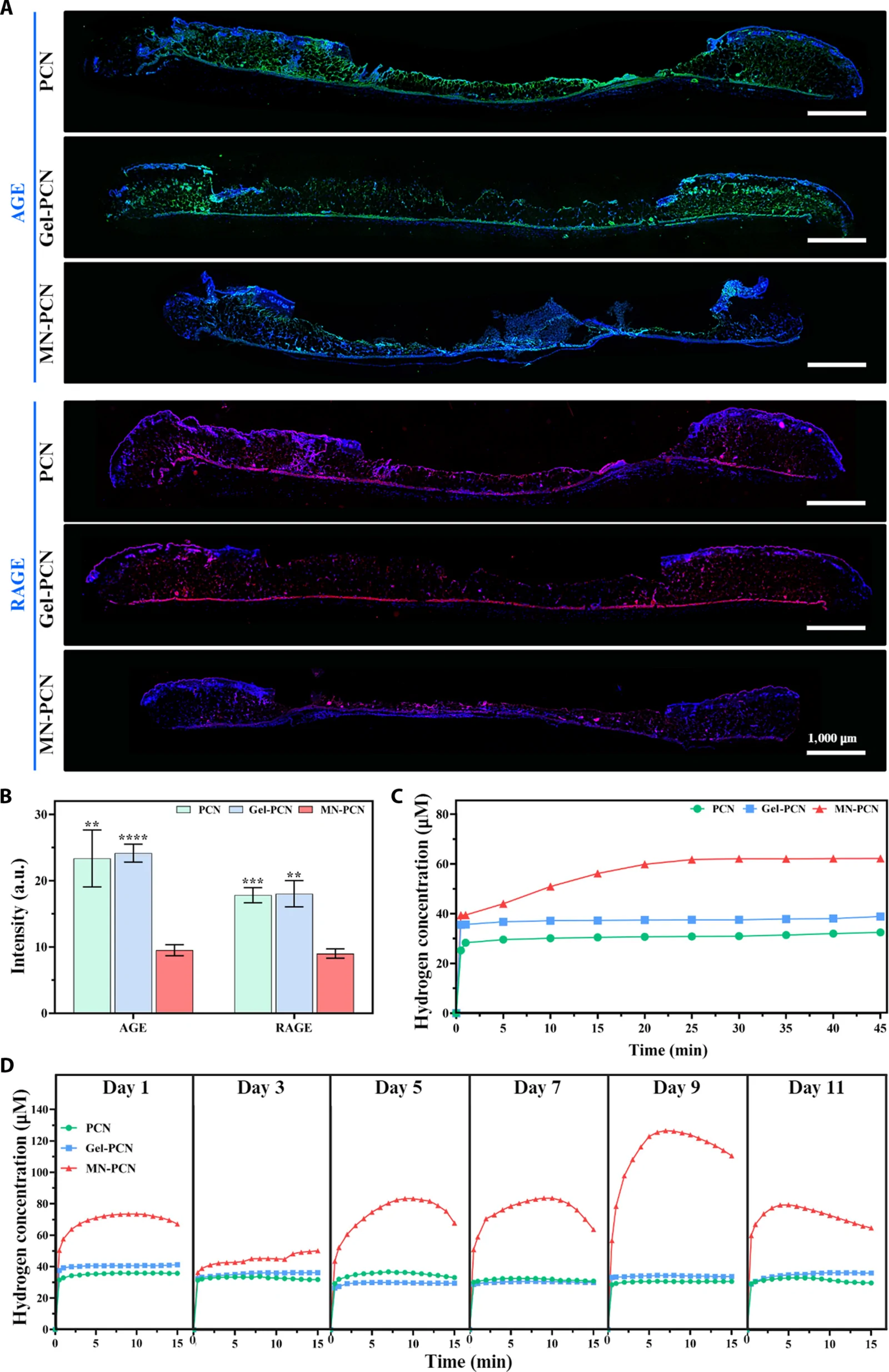
In vivo glucose depletion and hydrogen release efficacy of Pt@Pd-PCN222-NH_2_ (PCN), gel-PCN, and MN-PCN in diabetic burn wound healing. (A) Advanced glycation end product (AGE; green) and receptor for AGEs (RAGE; red) staining of wounded skin after 45 min of treatment with PCN powder, gel-PCN, or MN-PCN. Nuclei stained with 4′,6-diamidino-2-phenylindole (DAPI; blue). (B) Quantitative analysis of AGE and RAGE fluorescence intensities across treatment groups. (C) In vivo hydrogen concentration over a 45-min treatment period for the PCN, gel-PCN, and MN-PCN groups. (D) In vivo hydrogen concentration over an 11-d treatment period for the PCN, gel-PCN, and MN-PCN groups. Data represent mean ± SD (*n* = 3); significance: **P < 0.01; ***P < 0.001; ****P < 0.0001.

To elucidate the mechanisms underlying the systemic reconfiguration of a diabetic wound, we quantified the impact of MN-PCN on the metabolic–inflammatory axis. A hallmark of the diabetic pathological microenvironment is the accumulation of AGEs and their receptors (RAGEs), which sustain chronic inflammation. Immunofluorescence analysis of the wound tissues demonstrated that the MN-PCN system effectively disrupted this pathological cycle. Compared to the PCN-only group, MN-PCN treatment resulted in a 2.46-fold and a 1.98-fold reduction in AGE and RAGE levels, respectively (Fig. 5A and B). Crucially, the physical architecture of the microneedles played an important role in this metabolic remodeling. The presence of microneedle tips in the MN-PCN group resulted in an even greater reduction of AGE and RAGE intensities, achieving decreases of 2.54-fold and 2.01-fold compared to the needle-free gel-PCN group, respectively (Fig. 5B). This evidence confirms that the 500-μm microneedle structure is essential for delivering the catalytic centers into deep tissue to neutralize the hyperglycemic milieu.

The efficacy of this metabolism-driven therapy is further supported by the spatiotemporal dynamics of hydrogen evolution. In vivo monitoring via a hydrogen electrode revealed that the MN-PCN system reached a peak H_2_ concentration of 62.26 μM within 45 min, significantly exceeding the levels achieved by both PCN powder and gel-PCN groups, which remained below 40 μM (Fig. 5C). This value represents the local hydrogen concentration measured at the electrode tip positioned within the wound area, as detailed in Fig. S8, and thus reflects the H_2_ concentration available to the surrounding cells and tissues in the deep wound microenvironment. This enhanced H_2_ generation is attributed to the optimized capture of endogenous glucose as a sacrificial electron donor within the deep wound layers. Furthermore, the MN-PCN platform provided a sustained therapeutic window, maintaining H_2_ release for 11 d until the natural detachment of the microneedles (Fig. 5D). This prolonged H_2_ generation was supported by PCN released from the dissolving tips in the deep tissue layer, with the hydrogel backing serving as an additional reservoir. This localized, long-term production of H_2_ provides a continuous antioxidant and proangiogenic signal, which is superior to conventional external administration.

Collectively, these findings demonstrate that the MN-PCN system reconfigures the diabetic burn microenvironment through a synergetic physical–chemical approach. By integrating deep-tissue physical penetration with metabolism-driven H_2_ therapy, the platform effectively depletes pathological glucose and suppresses the AGEs/RAGE inflammatory signaling axis. This dual-remodeling strategy not only facilitates the removal of metabolic burdens but also provides a sustained biochemical foundation for tissue regeneration.

### In vivo metabolic remodeling and disruption of the AGEs/RAGE axis

**Fig. 6.**
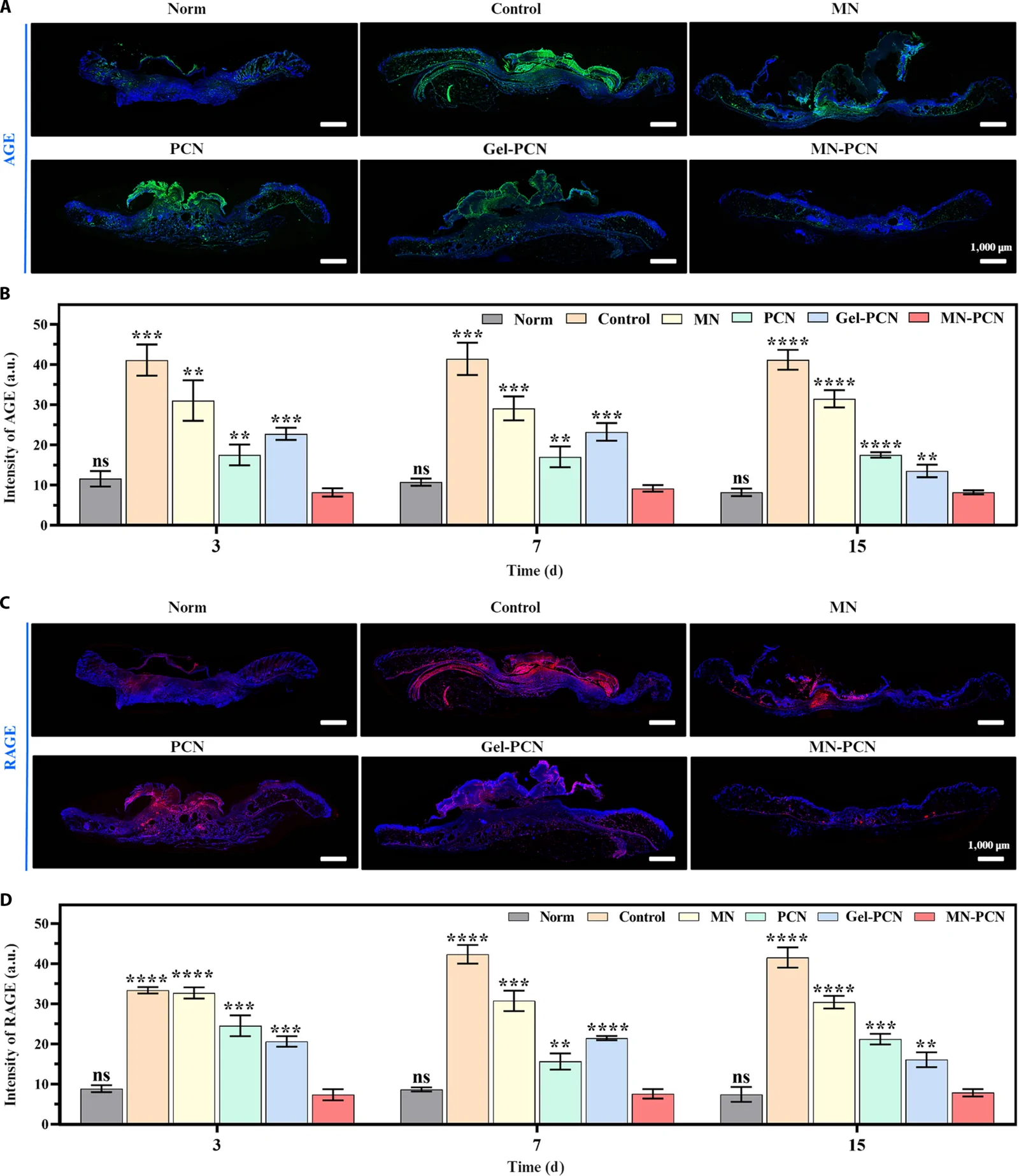
In vivo efficacy of MN-PCN in glucose depletion in diabetic burn wound healing. (A) Advanced glycation end product (AGE; green) staining in wounded skin after 15 d of treatment. Nuclei stained with 4′,6-diamidino-2-phenylindole (DAPI; blue). (B) Quantitative analysis of AGE intensity at 3, 7, and 15 d. (C) Receptor for AGEs (RAGE; red) staining in wounded skin after 15 d of treatment. Nuclei stained with DAPI (blue). (D) Quantitative analysis of RAGE intensity at 3, 7, and 15 d. Data represent mean ± SD (*n* = 3); significance: **P < 0.01; ***P < 0.001; ****P < 0.0001.

To investigate the efficacy of systemic metabolic remodeling driven by the MN-PCN system, we performed immunofluorescent staining for AGEs and RAGEs, which constitute the biochemical foundation of chronic inflammation in diabetic wounds. Skin samples were harvested at 3, 7, and 15 d posttreatment. Representative images and quantitative analysis of AGE staining revealed a progressive reduction in fluorescence intensity across all treatment groups (Fig. 6A and B and Fig. S9A and B). By day 15, the MN-PCN group exhibited a 1.65-fold lower AGE intensity compared to the needle-free gel-PCN group. This significant performance gap highlights the necessity of the 500-μm microneedle architecture in bypassing the eschar barrier to establish deep biochemical transmission channels, allowing the therapeutic PCN to access and deplete the pathological glucose in deeper tissue layers.

Consistent with the reduction in AGEs, RAGE expression was similarly suppressed by MN-PCN treatment (Fig. 6C and Fig. S9C and D). Quantitative assessment on day 15 showed that MN-PCN achieved a 2.05-fold greater reduction in RAGE intensity compared to the gel-PCN group (Fig. 6D). This localized metabolic reconfiguration effectively restored the AGEs/RAGE levels in diabetic tissue to a state comparable to that under normoglycemic physiological conditions. Notably, this localized remodeling was achieved without perturbing systemic blood glucose levels, which remained stable throughout the 15-d period (Fig. S3C), confirming that the therapeutic effect is specifically targeted to the wound microenvironment.

The robustness of this metabolic remodeling was further validated in an infected diabetic burn wound model, where the pathological microenvironment is exacerbated by *S. aureus* (Fig. S10A and B). The MN-PCN system still successfully reconfigured the hostile environment under these deteriorated conditions. The MN-PCN group exhibited a 3.03-fold decrease in AGE intensity relative to the control group and a 1.70-fold reduction compared to the gel-PCN group by day 15 (Fig. S10C). RAGE intensity followed a similar trend, showing a 2.26-fold decrease in the MN-PCN group compared to that in the gel-PCN group (Fig. S10D).

These results demonstrate that the MN-PCN system proactively reconfigures the diabetic burn microenvironment through a synergetic physical–chemical feedback mechanism. By coupling deep-tissue physical penetration with the metabolic conversion of pathological glucose into therapeutic H_2_, the system effectively neutralizes the metabolic basis of chronic inflammation. This integrated strategy not only facilitates the removal of pathological burdens but also reconstitutes the biochemical homeostasis required for tissue regeneration, maintaining its efficacy even in the presence of secondary infection.

### In vivo efficacy of MN-PCN in reducing ROS, promoting angiogenesis, and enhancing cell proliferation in diabetic burn wounds with or without infection

To evaluate the transition of the wound microenvironment from a hostile state to a reparative one, we analyzed the systemic effects of MN-PCN on oxidative stress, neovascularization, and tissue reconstruction. The integration of metabolic remodeling and physical feedback was quantified through ROS levels, capillary density (CD31), and cellular proliferation (Ki67) at 3, 7, and 15 d posttreatment.

**Fig. 7.**
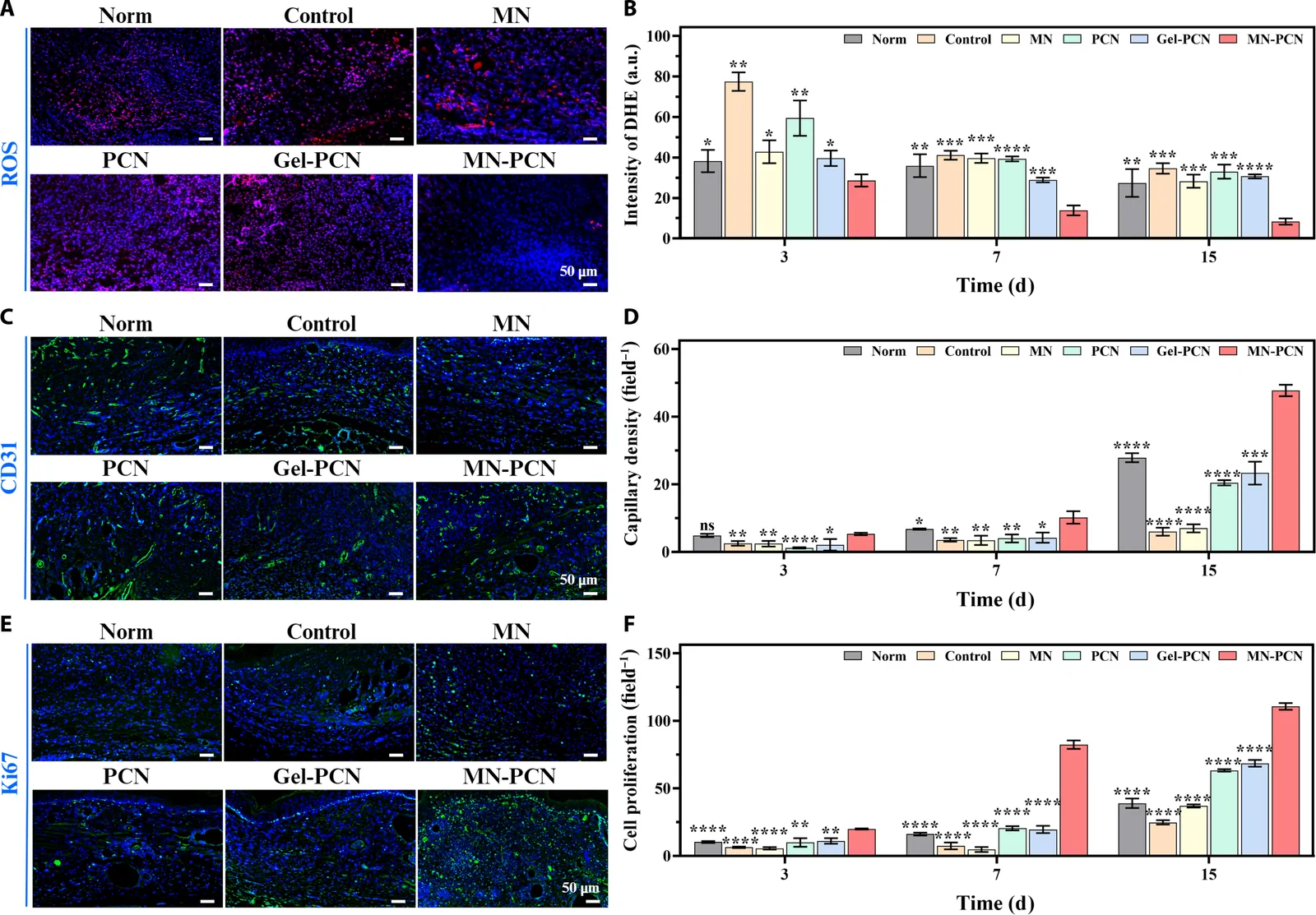
In vivo efficacy of MN-PCN in reducing reactive oxygen species (ROS), enhancing angiogenesis, and promoting cell proliferation in diabetic burn wound healing. (A) ROS (red) staining in wounded skin after 15 d of treatment. Nuclei stained with 4′,6-diamidino-2-phenylindole (DAPI; blue). (B) Quantitative analysis of ROS intensity at 3, 7, and 15 d. (C) CD31 (green) staining in wounded skin after 15 d of treatment. Nuclei stained with DAPI (blue). (D) Quantitative analysis of CD31 intensity at 3, 7, and 15 d. (E) Ki67 (green) staining in wounded skin after 15 d of treatment. Nuclei stained with DAPI (blue). (F) Quantitative analysis of Ki67 intensity at 3, 7, and 15 d. Data represent mean ± SD (*n* = 3); significance: *P < 0.05; **P < 0.01; ***P < 0.001; ****P < 0.0001.

After 3 d, the MN-PCN system achieved a 2.70-fold reduction in ROS production compared to the control group and was 1.38-fold more effective than the needle-free gel-PCN group. By day 15, this inhibitory effect was further amplified, reaching a 4.14-fold reduction in ROS levels relative to controls (Fig. 7A and B and Fig. S11A). This sustained antioxidant capacity, extending far beyond the typical acute inflammatory phase, is attributed to the microneedle-enabled deep delivery of PCN catalytic centers, which allows the CAT-like activity of Pt/Pd sites to decompose in situ generated H_2_O_2_ from photocatalytic glucose oxidation while the γ-PGA-CD hydrogel matrix concurrently provides supplementary ROS scavenging. This coordinated action ensures that the sustained H_2_ evolution proceeds without incurring secondary oxidative injury. The superior performance of MN-PCN over the needle-free gel-PCN group underscores that the microneedle architecture is essential for delivering the catalytic centers into deep tissue layers, where both the primary H_2_O_2_ generated during glucose oxidation and the environmental ROS can be effectively neutralized.

The coupling of physical architecture and biochemical signals was most evident in the stimulation of angiogenesis. By day 15, the MN-PCN group exhibited a 7.96-fold increase in capillary density compared to controls and a 2.05-fold increase over the gel-PCN group (Fig. 7C and D and Fig. S11B). This substantial enhancement validates the synergistic logic of the platform: while the 500-μm microneedle structure and adaptive PP-CA hydrogel provide a physical scaffold for cellular migration, the in situ generated H_2_ up-regulates proangiogenic signals such as VEGF, collectively accelerating neovascularization.

Correspondingly, cellular proliferation was markedly enhanced; MN-PCN treatment resulted in a 3.10-fold increase in Ki67-positive cells within the first 3 d and maintained a 1.62-fold superiority over gel-PCN by day 15, confirming the establishment of a robust reconstructive environment (Fig. 7E and F and Fig. S11C).

The robustness of this systemic reconfiguration was further confirmed in the hostile microenvironment of infected diabetic burn wounds. Despite the exacerbated pathology, the MN-PCN system sustained its regenerative momentum, yielding a 3.67-fold increase in capillary density and a 5.83-fold increase in regenerated cells compared to controls after 15 d (Fig. S12).

Overall, these results demonstrate that the MN-PCN platform does not merely offer isolated therapeutic functions but actively reconfigures the pathological landscape. By neutralizing oxidative burdens and providing integrated biomechanical–chemical feedback, the system effectively facilitates the transition from chronic nonhealing to comprehensive tissue restoration, even under complex infected conditions.

## Conclusion

In summary, we have developed a bio-integrated photocatalytic microneedle system (MN-PCN) that redefines the therapeutic strategy for diabetic burn wounds by transitioning from passive agent delivery to microenvironmental reconfiguration. The platform is constructed from an antimicrobial and antioxidant hydrogel (PP-CA) composed of γ-PGA-CD and ε-PLL-AD integrated with photocatalytic PCN and optimizes healing through 3 synergistic dimensions:

• Metabolism-driven H_2_ therapy: The integrated PCN catalytic centers exploit endogenous glucose as a sacrificial substrate to sustained H_2_ evolution, which up-regulates VEGF expression and promotes neovascularization, while simultaneously driving glucose depletion and suppressing the AGEs/RAGE signaling axis.
• Microenvironmental detoxification and antimicrobial protection: Leveraging the rationally designed Pt/Pd dual sites as a cascade nanozyme within the PCN framework, which exhibit coordinated SOD-, CAT-, and peroxidase-like activities, the system decomposes peroxides to water and oxygen. This nanozyme-enabled cascade, combined with the intrinsic ROS-scavenging capacity of the γ-PGA-CD matrix, effectively neutralizes peroxide intermediates and oxidative stress, thereby circumventing the secondary injury typically associated with uncontrolled photocatalysis. In parallel, the ε-PLL-AD component confers potent antimicrobial activity.
• Adaptive physical scaffolding: The self-adaptive microneedle architecture enables deep-tissue penetration and seamless biointerfacial adaptation and upon contact with wound exudate undergoes an in situ transition into a protective hydrogel barrier with superior wet-tissue adhesion. This physical shield not only safeguards against microbial invasion but also facilitates cellular infiltration, providing a permissive niche for tissue ingrowth.

Therefore, this platform, which combines catalytic nanozyme engineering, microneedle-mediated delivery, and adaptive hydrogel scaffolding, can be further adapted to other chronic wound types or metabolic disorders characterized by localized substrate overload and oxidative stress. The integrated functions of the MN-PCN system were systematically validated both in vitro and in vivo, demonstrating exceptional efficacy in neutralizing inflammatory circuits and restoring physiological homeostasis. By reestablishing both metabolic and structural equilibrium, this waste-to-treasure paradigm provides a sophisticated framework for the clinical management of complex chronic wounds and holds considerable translational potential for next-generation regenerative medicine.

## Materials and Methods

### Materials

γ-PGA (Mw = 1,000 to 15,000) was obtained from Sai Taisi Biological Technology Co., Ltd. (China), and ε-PLL (Mw = 2,000 to 5,000) was purchased from Aladdin (China). Cyclodextrin (CD) and adamantane were also sourced from Aladdin (China). Polydimethylsiloxane molds for microneedle patch fabrication were acquired from Taizhou Weixin Technology Co., Ltd. (China), with each patch designed as a 10 × 10 array measuring 260 μm × 260 μm × 500 μm (*W* × *L* × *H*). High-glucose Dulbecco’s modified Eagle medium (DMEM) cell culture medium and FBS were purchased from Gibco (UK). FBs and HUVECs were cultured in high-glucose DMEM supplemented with 10% FBS in an incubator maintained at 37 °C with 5% CO_2_.

### Fabrication and characterization of PCN

Photocatalytic PCN were synthesized by first preparing Hf-PCN222. This involved combining 323 mg of hafnium chloride with 163.4 mg of TCPP–COOH in 4.8 ml of trichloroacetic acid, followed by immersion in 80 ml of dimethylformamide (DMF) and heating at 120 °C for 5 h. The resulting mixture was centrifuged at 9,000 rpm and washed sequentially with DMF, absolute ethanol, and deionized water (ddH_2_O) and then dried to obtain Hf-PCN222.

For the synthesis of Pd@Hf-PCN222, 173 mg of PdCl_2_ and 149 mg of KCl were dissolved in 4 ml of ddH_2_O and mixed with 410 mg of Hf-PCN222 dispersed in 5 ml of ddH_2_O. This solution was heated at 60 °C until it turned rose-red and then centrifuged at 9,000 rpm to collect the sediment. The sediment was washed 5 times with ddH_2_O and dried at 100 °C for 12 h to obtain pure Pd@Hf-PCN222.

Next, 67.7 mg of Pd@Hf-PCN222 and 54 mg of *para*-aminobenzoic acid were dissolved in 12 ml of DMF and heated at 90 °C for 24 h. A solution containing 1.5 mg of H_2_PtCl_6_ and 1.5 mg of *para*-aminobenzoic acid was then added, and the reaction was continued for another 24 h. The mixture turned red, and pure PCN was obtained by centrifugation and washing with DMF, absolute ethanol, and ddH_2_O. The final PCN product was preserved for subsequent characterization.

### Fabrication and characterization of PP-CA hydrogel and MN-PCN

A 10 wt% γ-PGA hydrogel was prepared by dissolving 10 g of γ-PGA in ddH_2_O. Concurrently, a CD solution was formulated by dissolving 500 mg of CD in 10 ml of ddH_2_O, followed by the addition of 700 mg of 1-ethyl-3-(3-dimethylaminopropyl)carbodiimide. This mixture was stirred for 2 h and then allowed to react overnight at 4 °C. The CD solution was subsequently added to the γ-PGA hydrogel, stirred for an additional 2 h, and incubated overnight at 4 °C to facilitate cross-linking. The resulting γ-PGA-CD hydrogel was purified by dialysis for 2 d to remove excess CD.

The ε-PLL-AD hydrogel was synthesized following the same procedure, substituting absolute ethanol for ddH_2_O as the solvent. To form the PP-CA hydrogel, equal volumes of γ-PGA-CD hydrogel and ε-PLL-AD hydrogel were mixed and centrifuged at 20,000 rpm for 5 min.

Each 200 μl of PP-CA hydrogel, with or without 150 μg ml^−1^ PCN, was dispensed into polydimethylsiloxane microneedle molds, centrifuged at 3,000 rpm for 5 min, and dried at 40 °C for 12 h. After drying, the microneedles were demolded to obtain MN and MN-PCN patches. Additionally, gel-PCN patches, consisting of PP-CA hydrogel loaded with PCN but without needle tips, were fabricated using the same procedure.

The morphology of MN-PCN was characterized using a Nikon Eclipse E100 microscope (Nikon Corporation, Japan). Additionally, MN-PCN was placed in a sealed tank at 25 °C and 75% humidity, and morphological changes were recorded at specific time points using a Leica DMi8 fluorescent microscope (Germany).

### Self-adaptive properties of PP and PP-CA hydrogel

FBs were transduced with GFP to visualize cellular behavior in co-culture experiments. These GFP-labeled FBs were then co-cultured with microneedle patches fabricated from PP hydrogel or PP-CA hydrogel. The co-culture was maintained for 1 and 3 d to assess cellular interaction and integration with the hydrogel matrices. After co-culture, representative images of the FBs were captured using a Leica DMi8 microscope (Germany).

### The in vitro glucose depletion and hydrogen release efficacy of PCN and MN-PCN

Each 2 ml of high-glucose DMEM (3.2 g l^−1^) was placed into a well of a 12-well plate and divided into 3 groups: control, PCN, and MN-PCN. The plates were irradiated with a 300-W Xe lamp (*λ* ≥ 420 nm, 0.1 W cm^−2^) for 15, 30, 45, or 60 min. After irradiation, samples from each well were collected for glucose concentration analysis using an *O*-toluidine-based glucose assay kit (Beyotime, China) according to the manufacturer’s instructions.

Separately, PCN solution or one patch of MN-PCN was placed in a sealed bottle containing 10 ml of ddH_2_O with 4.5 g l^−1^ glucose. Each bottle was then irradiated with a 300-W Xe lamp (*λ* ≥ 420 nm, 0.1 W cm^−2^) for 3 min. Hydrogen release was quantitatively measured by immersing a hydrogen electrode into the solution, and stable readings were recorded for analysis.

### In vitro antimicrobial analysis

To evaluate the antimicrobial effect of MN-PCN, *S. aureus* diluted to 10^3^ CFU ml^−1^ was applied. The bacterial suspension was seeded onto agarose plates, which were then divided into 4 groups: control (25 μl of sterile water), MN extract, PCN solution (15 μg ml^−1^), and MN-PCN extract. The plates were incubated for 48 h, after which representative images were collected and the diameters of inhibition zones were quantified.

### CCK-8 assay

The cytotoxicity of PCN was evaluated by seeding HUVECs into a 96-well plate at a density of 3,000 cells per well and co-culturing them with varying concentrations of PCN solution (0, 5, 10, 15, 20, 25, and 30 μg ml^−1^) for 3 d. During the co-cultivation period, the cells were irradiated daily with a 300-W Xe lamp (*λ* ≥ 420 nm, 0.1 W cm^−2^) for 3 min. Cell viability was assessed using a CCK-8 assay following the manufacturer’s instructions. After a 2-h incubation with the CCK-8 solution, absorbance at 450 nm was measured using a SpectraMax iD3 microplate reader (Molecular Devices LLC, San Jose, CA, USA).

To evaluate the antioxidant efficacy, MN and MN-PCN patches were immersed in 5 ml of complete cell culture medium for 3 h to obtain the extracts. FBs and HUVECs were seeded into 96-well plates at a density of 3,000 cells per well and co-cultured with either 10 μl of extract plus 90 μl of complete cell culture medium or 100 μl of complete cell culture medium (control) for 3 d. Each plate was subjected to daily 3-min irradiation with a 300-W Xe lamp (*λ* ≥ 420 nm, 0.1 W cm^−2^). On the third day, cells were either exposed to 3 mM H_2_O_2_ for 30 min before performing the CCK-8 assay for 2 h or directly subjected to the CCK-8 assay without H_2_O_2_ exposure. The absorbance at 450 nm was measured and recorded for quantification.

### In vitro antioxidant analysis

Raw264.7 cells were seeded into 6-well plates and divided into 4 groups: MN extract, PCN solution (15 μg ml^−1^), MN-PCN extract, and no treatment (control). After 24 h of co-cultivation, an ROS assay (Beyotime, China) was performed according to the manufacturer’s protocol to assess ROS levels in the cells. Fluorescent images of the cells in each group were captured using a Leica DMi8 microscope (Germany). Subsequently, cells from each group were analyzed by flow cytometry (Beckman Coulter, USA) to quantify the proportion of fluorescent cells, providing a quantitative measure of ROS reduction.

### Live/dead cell double staining

FBs and HUVECs were seeded at a density of 4 × 10^5^ cells per well into 6-well plates and divided into 4 groups: 15 μg ml^−1^ PCN solution, one patch of MN, MN-PCN, and no treatment (control). Each day, the cells were irradiated with a 300-W Xe lamp (*λ* ≥ 420 nm, 0.1 W cm^−2^) for 3 min. After 3 d of incubation, a calcein-AM/propidium iodide live/dead staining assay (300 μl) was added to each well and incubated for 15 min at 37 °C with 5% CO_2_. Representative images of FBs and HUVECs in each group were captured using a Leica DMi8 fluorescent microscope (Germany).

### In vitro cellular migration analysis

To assess the migration efficacy of MN-PCN on HUVECs, a Transwell assay was conducted. Each lower chamber of a 24-well Transwell plate received 750 μl of complete cell culture medium, while 2 × 10^4^ HUVECs in 200 μl of medium containing 1% FBS were placed in the upper chamber. The HUVECs were divided into 4 groups and co-cultured with corresponding materials by adding 100 μl of cell culture medium (control group), MN extract, 15 μg ml^−1^ PCN solution, or MN-PCN extract into the upper chamber for 24 h. After fixation with paraformaldehyde for 15 min, Hoechst (Beyotime, China) was applied for staining. Nonmigratory cells were removed from the upper surface of the Transwell membrane, and only migratory HUVECs remained, which were then photographed using a fluorescent microscope (Leica DMi8, Germany).

The scratch test was used to examine the migration of FBs. Cells were seeded into 6-well plates and serum-starved for 12 h. Uniform scratches were made using a pipette tip. Subsequently, FBs were divided into 4 treatment groups similar to the Transwell assay. At specific time points, representative images of cells in each group were obtained using a Nikon Eclipse TS100 microscope (Nikon Corporation, Japan).

### Tube formation analysis

Each well of a 96-well plate was coated with 50 μl of Matrigel and incubated at 37 °C with 5% CO_2_ for 30 min to allow gelation. Meanwhile, 1.5 × 10^4^ HUVECs were suspended in 100 μl of cell culture medium containing 1% FBS (control group), MN extract, or MN-PCN extract, obtained by immersing MN and MN-PCN in cell culture medium with 1% FBS for 3 h. The cell suspension was then added to the Matrigel-coated wells and incubated at 37 °C with 5% CO_2_ for 4 h to facilitate cell–matrix interactions. Representative images were captured using a Nikon Eclipse TS100 microscope (Nikon Corporation, Japan), followed by quantitative analysis of tube formation.

### RT-qPCR analysis

FBs and HUVECs were seeded at a density of 1.5 × 10^5^ cells per well into 6-well plates. After 24 h of co-cultivation with MN extract, PCN solution (15 μg ml^−1^), MN-PCN extract, or no treatment (control), RNA was isolated from the cells. The mRNA expression levels of RAGEs in FBs and both RAGEs and VEGF in HUVECs were analyzed using RT-qPCR. Glyceraldehyde 3-phosphate dehydrogenase (GAPDH) was used as the housekeeping gene.

### Establishment and treatment of diabetic burn and infected diabetic burn wound model

All experimental protocols were approved by the Institutional Animal Care and Experiment Committee of Shanghai Jiaotong University School of Medicine, ensuring adherence to ethical standards (Approval No.: SH9H-2024-A1007-1). To establish the diabetic burn wound model, 60 diabetic (*db/db*) mice (8 weeks old) and 12 BKS mice (8 weeks old) were used. Each mouse was anesthetized and shaved, and a circular 1-cm-diameter burn wound was created on their back using a controllable electric soldering iron. Eschars formed after 24 h were removed under anesthesia. The 60 diabetic mice were randomly divided into 5 groups and treated with PCN powder (30 μg), MN, gel-PCN, MN-PCN, or PBS (control). BKS mice served as normoglycemic controls and were treated with PBS.

For the infected diabetic burn wound model, a similar procedure was followed using 25 diabetic mice (8 weeks old) and 5 BKS mice (8 weeks old). After eschar removal, wounds were inoculated with 50 μl of *S. aureus* (10^6^ CFU ml^−1^). Each mouse was housed individually with sufficient food and water. Mice in different groups were irradiated with a 300-W Xe lamp (*λ* ≥ 420 nm, 0.05 W cm^−2^) for 15 min every other day and photographed after 0, 1, 3, 5, 7, 9, 11, 13, and 15 d of treatment.

### In vivo short-time glucose depletion and hydrogen release

To assess the short-term efficacy of MN-PCN in glucose depletion and hydrogen release, 12 diabetic mice (8 weeks old) were anesthetized, shaved, and subjected to a 1-cm-diameter deep burn wound on their backs. After tangential excision, mice were randomized into 3 groups and treated with 30 μg of PCN powder, a gel-PCN patch, or a MN-PCN patch. Each mouse was then exposed to a 300-W Xe lamp (*λ* ≥ 420 nm, 0.05 W cm^−2^) to activate the treatment, and hydrogen release was monitored using a hydrogen electrode. After 45 min, mice were euthanized, and skin tissues from the wound area were harvested for immunofluorescence analysis using antibodies against AGEs and RAGEs.

For long-term hydrogen release monitoring, a similar wound model was created on diabetic mice, supplemented with a 1-mm circular wound for electrode insertion. After randomly dividing mice into 3 groups with corresponding treatments, the hydrogen electrode was positioned in the 1-mm wound to measure hydrogen release when the 300-W Xe lamp (*λ* ≥ 420 nm, 0.05 W cm^−2^) irradiated the burn wound area. This process was repeated for 15 min bi-daily over 11 d to track healing progression.

### In vivo cytotoxicity analysis

After 15 d of treatment, mice in the gel-PCN and MN-PCN groups were euthanized. Vital organs, including the heart, kidneys, and liver, were carefully excised and immediately immersed in 4% paraformaldehyde for a 24-h fixation period. Subsequently, the fixed tissues underwent a series of dehydration steps, were cleared, and were then embedded in paraffin wax blocks. Sections from these blocks were prepared for H&E staining to assess histopathological changes.

### In vivo antimicrobial analysis

To confirm the establishment of the infected diabetic burn wound model, a representative BKS mouse was euthanized 48 h postapplication of *S. aureus*. The skin tissue from the wound site was excised and briefly immersed in 1 ml of sterile water for 10 s. Subsequently, 100 μl of this solution was uniformly spread onto an agarose plate, which was then incubated for 36 h at 37 °C. To further test the antimicrobial efficacy of the materials, 1 μl of wound exudate from each mouse in different groups after 7 d of treatment was collected using an inoculation ring and diluted 10^4^ times with sterile water. Each 100 μl of the diluted sample was then spread onto separate agarose plates and incubated at 37 °C for 36 h to observe bacterial colony formation.

### In vivo wound healing, ROS reduction, and long-term glucose depletion analysis

To quantify the wound healing rate, wound areas were photographed at the indicated time points (0, 1, 3, 5, 7, 11, and 15 d) using a digital camera with a fixed distance and constant lighting conditions. For each wound, the wound boundary was manually traced using the ImageJ software (National Institutes of Health, USA), and the pixel area was measured. The wound healing rate (percentage) was calculated as wound healing rate (%) = [(*A*_0_ − *A_t_*)/*A*_0_] × 100%, where *A*_0_ represents the initial wound area measured immediately after treatment (day 0) and *A_t_* represents the wound area at each subsequent time point (day *t*).

Mice with burn wounds were euthanized after 3, 7, and 15 d of treatment, and mice with infected burn wounds were euthanized after 15 d of treatment. All skin tissues from the wound area were excised and immediately fixed with 4% paraformaldehyde. After 24 h, the tissues were dehydrated, cleared, and embedded in paraffin blocks. Sections were cut and subjected to immunohistochemical staining for ROS, CD31, Ki67, AGEs, and RAGEs. Additionally, H&E and Masson trichrome staining were performed to examine the healing process.

### Statistical analysis

All experimental outcomes were consistently replicated in at least 3 independent trials and are presented as mean ± standard deviation. Statistical comparisons between 2 distinct experimental conditions were conducted using an unpaired 2-tailed *t* test. Quantitative analyses of wound areas, angiogenesis, and immunofluorescence intensities were performed using the ImageJ software (National Institutes of Health, USA) via the analysis of at least 3 randomly selected fields per wound section. For immunofluorescence quantification, background fluorescence was subtracted by measuring a background region in ImageJ, and corrected total fluorescence was calculated as integrated density minus (area × mean background fluorescence). Comprehensive statistical evaluations were carried out with GraphPad Prism (version 9.0, GraphPad Software, San Diego, CA, USA), ensuring rigorous data integrity. Significance levels were denoted as follows: *P < 0.05, **P < 0.01, ***P < 0.001, and ****P < 0.0001, indicating statistically significant variances, while “ns” represents a lack of statistical significance.

## Ethical Approval

All experimental protocols were approved by the Institutional Animal Care and Experiment Committee of Shanghai Jiaotong University School of Medicine, ensuring adherence to ethical standards (Approval No.: SH9H-2024-A1007-1).

## Data Availability

The authors declare that the main data supporting the findings of this study are available within the article and its Supplementary Materials. Extra data are available from the corresponding authors upon request.
