# Supplementary material for: Waste-to-Treasure: Nanozyme-Integrated Photocatalytic Microneedles Reconfigure Diabetic Burn Wounds via Endogenous Glucose-to-H2 Conversion and Adaptive Hydrogel Scaffolding
Source: Research (Wash D C). 2026 Sep 23;9:1403. doi: 10.34133/research.1403 (PMC13598330; doi:10.34133/research.1403)
Supplement: Supplementary file 1 [file research.1403.f1.zip › Supplementary Information-clean.docx]

Supporting Information for

**Waste-to-Treasure: Nanozymes-Integrated Photocatalytic Microneedles Reconfigure Diabetic Burn Wounds via Endogenous Glucose-to-H₂ Conversion and Adaptive Hydrogel Scaffolding**

*Pei Wang, Xiaofeng Wang, Yating Yang, Zhen Gao, Xiaoxi Lin, Xiansong Wang*

(P. Wang and X-F. Wang, contributed equally to this work)

P. Wang, Y-T. Yang, Dr. Z. Gao, Prof. X-X. Lin, Prof. X-S. Wang

Department of Plastic and Reconstructive Surgery, Shanghai Key Laboratory of Tissue Engineering, Shanghai Ninth People’s Hospital, Shanghai Jiao Tong University School of Medicine, Shanghai 200011, China. E-mail: wonderluis@sjtu.edu.cn (X-S. W.), [linxiaoxi@126.com](mailto:linxiaoxi@126.com) (X-X. L), shgaozhen@126.com (Z. G.)

Prof. X-F. Wang

Excellent Science and Technology innovation Group of Jiangsu Province, College of Environmental Science, Nanjing Xiaozhuang University, Nanjing 211171, China.

.

**Fig. S1. Fabrication and properties of PP-CA hydrogel.** (A) Schematic of PP-CA hydrogel fabrication. (B) Self-healing ability of PP-CA hydrogel. (C) Self-healing demonstration with Indocyanine Green staining. (D) Enhanced self-adaptation of PP-CA hydrogel, shown by fibroblast (FB) ingrowth after 3 days of co-culture with a microneedle patch. (E) Glucose depletion by PCN and MN-PCN. (F) Hydrogen release profiles of PCN and MN-PCN. (G) Antibacterial efficacy against *S. aureus* of MN, PCN, and MN-PCN. (H) Quantification of bacteriostatic zone diameters for each treatment group. Data represent mean ± SD (n = 3); significance: *****p < 0.05, ******p < 0.01, *******p < 0.001, ********p < 0.0001.

| **Gene** | **Primer** | **Sequence (5’-3’)** | **Length** |
| --- | --- | --- | --- |
| RAGE (FB) | Forward | CCAGAGGGCATTCAGCTGTT | 20 |
|  | Reverse | AGGGTGCACCATCCTTTATCC | 21 |
| RAGE (HUVECs) | Forward | GACAGTGTGGCTCGTGTCCT | 20 |
|  | Reverse | CTGGCTTCCCAGGAATCTGGT | 21 |
| VEGF (HUVECs) | Forward | GTACATCTTCAAGCCATCCTGTG | 23 |
|  | Reverse | GTTTGATCCGCATAATCTGCATG | 23 |

**Table S1. Summary of primer sequences.**

**Fig. S2. *In vivo* degradation of MN-PCN microneedles.** Photographic series illustrating the degradation of MN-PCN microneedles over time *in vivo*.

**Fig. S3. *In vivo* biocompatibility and systemic effects of MN-PCN.** (A) H&E staining of heart, kidney, and liver tissues from mice treated with gel-PCN and MN-PCN after 15 days. (B) Weight changes in mice with burn wounds across different treatment groups. (C) Blood glucose levels in mice with burn wounds across treatment groups. Data represent mean ± SD (n = 3).

**Fig. S4. Histological analysis of wound healing in diabetic mice.** (A) H&E staining of skin tissues from treated diabetic mice after 15 days. (B) Masson trichrome staining of skin tissues from treated diabetic mice after 15 days.

**Fig. S5. Antibacterial efficacy and systemic effects of MN-PCN in infected diabetic wounds.** (A) Bacterial colony images from infected wounds in BKS mice after 48 hours of *S. aureus* application. (B) Schematic of the *in vivo* antibacterial experiment procedure. (C) Bacterial colony images from infected wounds after 7 days of treatment. (D) Weight changes in mice with *S. aureus*-infected wounds across treatment groups. (E) Blood glucose levels in mice with *S. aureus*-infected wounds across treatment groups. Data represent mean ± SD (n = 3); significance: *****p < 0.05, ******p < 0.01, *******p < 0.001, ********p < 0.0001.

**Fig. S6. Therapeutic efficacy of MN-PCN on infected diabetic wounds.** (A) Representative images of BKS and diabetic mice with *S. aureus*-infected burn wounds treated with PBS, MN, PCN powder, gel-PCN, or MN-PCN over 0, 1, 3, 5, 7, 11, and 15 days. Microneedle patches were left intact until natural detachment. (B) Quantitative analysis of wound area across treatment groups over time. Data represent mean ± SD (n = 3); significance: *****p < 0.05, ******p < 0.01, *******p < 0.001, ********p < 0.0001.

**Fig. S7. Histological analysis of infected wound healing in diabetic mice.** H&E (A) and Masson trichrome (B) staining of skin tissues from diabetic mice with *S. aureus*-infected wounds after 15 days of treatment.

**Fig. S8. Experimental setup for *in vivo* hydrogen release measurement.** (A) Creation of a 1 cm diameter burn wound on the back of a mouse, followed by a 1 mm circular wound beneath it. (B) Application of an MN-PCN patch to the burn wound. (C) Irradiation of the wound area with a 300 W Xe lamp (λ ≥ 420 nm, 0.05 W cm^-^²) for 15 minutes while inserting a hydrogen electrode into the small wound to detect hydrogen concentration.

**Fig. S9. AGEs and RAGE staining in diabetic wound healing.** (A, B) Representative images of AGE (green) staining in wounded skin tissues after 3 and 7 days of treatment. Nuclei stained with DAPI (blue). (C, D) Representative images of RAGE (red) staining in wounded skin tissues after 3 and 7 days of treatment. Nuclei stained with DAPI (blue).

**Fig. S10. Glucose depletion in infected diabetic wound healing with MN-PCN.** (A, C) Representative images and quantification of AGE (green) staining in wounded skin tissues after 15 days of treatment. Nuclei stained with DAPI (blue). (B, D) Representative images and quantification of RAGE (red) staining in wounded skin tissues after 15 days of treatment. Nuclei stained with DAPI (blue). Data represent mean ± SD (n = 3); significance: *****p < 0.05, ******p < 0.01, *******p < 0.001, ********p < 0.0001.

**Fig. S11. ROS reduction, angiogenesis enhancement, and cell proliferation in diabetic wound healing with MN-PCN.** (A) ROS (red) staining in wounded skin tissues after 3 and 7 days of treatment. Nuclei stained with DAPI (blue). (B) CD31 (green) staining in wounded skin tissues after 3 and 7 days of treatment. Nuclei stained with DAPI (blue). (C) Ki67 (green) staining in wounded skin tissues after 3 and 7 days of treatment. Nuclei stained with DAPI (blue).

**Fig. S12. Angiogenesis and cell proliferation in infected diabetic wound healing with MN-PCN.** (A, B) Representative images and quantification of CD31 (green) staining in wounded skin tissues after 15 days of treatment. Nuclei stained with DAPI (blue). (C, D) Representative images and quantification of Ki67 (green) staining in wounded skin tissues after 15 days of treatment. Nuclei stained with DAPI (blue). Data represent mean ± SD (n = 3); significance: *****p < 0.05, ******p < 0.01, *******p < 0.001, ********p < 0.0001.
